# An Autonomous Sleep-Stage Detection Technique in Disruptive Technology Environment

**DOI:** 10.3390/s24041197

**Published:** 2024-02-12

**Authors:** Baskaran Lizzie Radhakrishnan, Kirubakaran Ezra, Immanuel Johnraja Jebadurai, Immanuel Selvakumar, Periyasami Karthikeyan

**Affiliations:** 1 Department of Computer Science and Engineering, Karunya Institute of Technology and Sciences, Coimbatore 641114, India; radhakrishnanbl@karunya.edu.in (B.L.R.); immanueljohnraja@karunya.edu (I.J.J.); 2 Department of Computer Science and Engineering, Grace College of Engineering, Coimbatore 628005, India; ekirubakaran@gracecoe.org; 3 Department of Electrical and Electronics Engineering, Karunya Institute of Technology and Sciences, Coimbatore 641114, India; immanuel@karunya.edu; 4 School of Computer Science and Engineering, RV University, Bengaluru 560059, India

**Keywords:** single-channel EEG, machine learning, sleep-stage classification, particle swarm optimisation (PSO), XGBoost, sleep monitoring, sleep staging, AASM, EEG, sustainable technologies

## Abstract

Autonomous sleep tracking at home has become inevitable in today’s fast-paced world. A crucial aspect of addressing sleep-related issues involves accurately classifying sleep stages. This paper introduces a novel approach PSO–XGBoost, combining particle swarm optimisation (PSO) with extreme gradient boosting (XGBoost) to enhance the XGBoost model’s performance. Our model achieves improved overall accuracy and faster convergence by leveraging PSO to fine-tune hyperparameters. Our proposed model utilises features extracted from EEG signals, spanning time, frequency, and time–frequency domains. We employed the Pz-oz signal dataset from the sleep-EDF expanded repository for experimentation. Our model achieves impressive metrics through stratified-K-fold validation on ten selected subjects: 95.4% accuracy, 95.4% F1-score, 95.4% precision, and 94.3% recall. The experiment results demonstrate the effectiveness of our technique, showcasing an average accuracy of 95%, outperforming traditional machine learning classifications. The findings revealed that the feature-shifting approach supplements the classification outcome by 3 to 4 per cent. Moreover, our findings suggest that prefrontal EEG derivations are ideal options and could open up exciting possibilities for using wearable EEG devices in sleep monitoring. The ease of obtaining EEG signals with dry electrodes on the forehead enhances the feasibility of this application. Furthermore, the proposed method demonstrates computational efficiency and holds significant value for real-time sleep classification applications.

## 1. Introduction

The twenty-first century is witnessing a significant spurt in patients having sleep-related issues. The growing percentage of sleep-related problems and their grave association with cardiovascular, immunological, metabolic, and cognitive dysfunctions are a severe concern of the present technologically advanced world. Researchers address this trend as a severe public health issue [1]. Among the sleep-tracking approaches, the gold standard for estimating sleep is polysomnography (PSG). Although there are numerous advantages to using PSG in clinical sleep assessment, the high cost limits its accessibility to many people. Furthermore, factors such as an unusual sleeping environment, restricted privacy in the sleep lab, skin irritation due to adhesion from electrodes, multiple leads linked to the person, and crossing wires, could impede sleep. The factors cited above lower the accuracy of sleep recordings [2].

There has been a constant evolution in adopting home-based sleep monitoring systems, particularly in this COVID era [3]. Technology is the critical enabler in designing robust, convenient, and low-cost home-based sleep monitoring systems using single-channel (SC) EEG (dry or wet) [4,5]. In-home-PSG is a valid alternative to in-sleep lab PSG without compromising the gold standard aspect. In-home scenarios, Raspberry Pi (R-Pi) is a popular choice to capture EEG signals; it facilitates designing low-cost and affordable sleep monitoring systems [6,7]. The captured signals are processed and classified as distinct sleep stages using automated sleep-stage classification (ASSC) systems.

In ASSC systems, machine learning is crucial in classifying the sleep stages. Many researchers have presented several methods for ASSC employing single-channel EEG. A reliable and accurate ASSC provides healthcare providers with efficient and meaningful information [8]. Even though single-channel provides cheap ASSC schemes, designing a practical, accurate ASSC remains challenging. The real challenge is to develop an efficient and robust ASSC system that preserves crucial discriminatory information for improved classification accuracy. Consequently, systematic electroencephalogram (EEG) signal handling is mandatory for an efficient automated EEG signal classification [9]. It has led to the development of many computer-based single-channel EEG analysis and classification approaches, discussed in the related work Section 2. The typical ASSC systems using machine learning follow the following workflow:

- Data collection or extraction;
- Pre-processing (filtering and removing unnecessary data points);
- Converting the signal into epochs;
- Feature extraction;
- Classification.

Extreme gradient boosting (XGBoost) is a sophisticated, robust, and powerful algorithm for predictive modelling [10]. The versatile nature of XGBoost enables it to be a better candidate for deployment in home-based low-resource scenarios [11,12]. Creating a classification model using XGBoost is very simple but uses multiple hyperparameters. Incorrect hyperparameter settings will result in poor prediction of results. Hyperparameter tuning is inevitable in designing a good-performing model. It is hard to decide on the ideal group of hyperparameters to achieve optimal performance [13]. In a tree-based model like XGBoost, when generating predictions, hyperparameters play the decision variable’s role at every node, and the numeric thresholds are applied to determine whether to move the left or right branch. Hence, this indicates the need to optimise the hyperparameters in order to improve the classification accuracy [14,15]. Particle swarm optimisation (PSO) is a widely used approach to optimise targets using population behaviour. This research proposes the PSO–XGBoost model, which blends swarm intelligence optimisation with XGBoost to improve the EEG signal’s sleep stage detection accuracy. The following are the key contributions of the proposed work:

- A PSO–XGBoost model is proposed and implemented where PSO is used to optimise the hyperparameters of XGBoost to enrich the overall accuracy during the classification task.
- To assess the proposed PSO–XGBoost method’s performance, the overall metrics and kappa values in each fold are compared to the existing approaches.

The remainder of this article is structured as follows. Section 2 explains the recent research using single-channel EEG. Section 3 discusses the dataset employed in experiments and the list of features extracted. Section 4 illustrates the experimental details, outcomes, and comparison with the existing works. Section 5 concludes the proposed PSO–XGBoost and its outcome.

## 2. Related Work

Sleep researchers have proposed diverse approaches to automate sleep scoring (sleep stage classification). Several signal processing methods and machine learning algorithms extract meaningful information from EEG signals. Generally, the performance of the machine learning approach depends on the extracted features from the data. According to the literature, time-domain, frequency-domain, and time–frequency-domain features are commonly extracted from the data and fed into the classifiers for detecting the specific sleep stages [16]. Based on the approaches, the existing works related to single-channel are grouped and presented in this section.

Various studies have highlighted the value of extracting characteristics from the EEG signal’s ensemble empirical mode decomposition (EEMD) domain. The EMD converts the nonlinear and non-stationary nature of the EEG signal into a finite set of intrinsic mode functions (IMFs). The EEMD method makes every IMF physically meaningful by quantifying the instantaneous amplitude and frequency. In [17,18,19], the authors extracted statistical moment and adaptive-noise-based features from the EMD domain. They used the classifiers Adaboost, Bagging, and RUSboost and achieved an accuracy of 90.11%, 90.69%, and 83.49%, and kappa values of 0.89, 0.89, and 0.84, respectively. In a similar approach proposed in [20], the authors obtained statistical, nonlinear, and time-domain features from the subbands of the EMD domain of the raw EEG signal. Then, the XGBoost model was trained and tested using the extracted features and attained 92.2% accuracy and 0.88 kappa.

A hidden Markov model (HMM)-based refinement has been employed and tested by some researchers to enhance the prediction accuracy of the sleep stage. Jiang et al. designed a scheme employing HMM-based refining to discover the sleep stage change probability and the classifier’s confusion matrix to train the random forest (RF) classifier. The sub-bands like δ (delta), θ (theta), α (alpha), σ (sigma), β (beta), γ (gamma), and K-complex characteristic waves are the typical components of EEG signals. Different sub-band waves in the EEG correspond to varying stages of sleep. This study decomposed the EEG signal into eight sub-bands and seven IMFs from the EMD domain. The statistical features are computed from the raw EEG, and the statistical, spectral, and fractal features are extracted from both the sub-bands and IMFs. After HMM refinement, the five-class classification using an RF classifier achieved an overall accuracy of 88.3% and 0.81 kappa [21]. A similar method proposed in [22] used nonlinear, cepstral, wavelet, time, and auto-regressive features using an RF classifier and obtained an accuracy of 81.86% and 0.74 kappa, respectively.

Researchers use a tunable-Q factor wavelet-transform (TQWT), a data-adaptive and flexible signal decomposition approach to process oscillatory signals. The authors of [23] decomposed the signal into six sub-bands with the help of TQWT. Next, statistical moment features are extracted from the sub-bands, fed into the Bagging classifier, and attained an accuracy of 78.95% and 0.82 kappa. The author, Hassan et al. [24], proposed an RF-based sleep stage classification system. First, they extracted spectral features from the EEG signal decomposed using TQWT. Next, the spectral features were fed into the RF classifier and achieved 91.50% accuracy and 0.86 kappa in five-stage classification.

A hybrid signal decomposition approach comprising EEMD and TQWT is proposed in [25]. First, the raw signals are decomposed using EEMD, and only the first two IMFs are selected. Then, the selected IMFs and the raw signal are decomposed by employing TQWT. TQWT offers the option to change the Q value in their filters to make the components more focused in the appropriate frequency bands. The first two IMFs have more fluctuation; hence, they possess more information that is valuable for classification. The most crucial EEG information with the most significant magnitude can be collected from the first four wavelet components. A total of 20 features were extracted: five statistical features from the four selected bands. Finally, the classification was performed using the bagging classifier and achieved an accuracy of 89.37% and 91.29% using the sleep-EDFx and sleep-EDF datasets [25].

More recently, researchers extracted features only from the sub-bands δ (delta), θ (theta), α (alpha), σ (sigma), β1 (beta 1), β2 (beta 2), γ1 (gamma 1), γ2 (gamma 2), and K-complex waves of EEG signals [26,27,28]. The authors employed this scheme because, during N2 and SWS sleep, the dominant waves are σ and δ. Similarly, in W and REM, the dominant waves are α and β, whereas in N1, the dominant wave is θ. Furthermore, in [27], authors mentioned that γ (30–49.5 Hz) wave impacts the classification of sleep stages and that the absence of the γ wave leads to a notable decline in performance. Additionally, after 60 years of age, the α wave slows down, and the β wave rises with age but falls beyond the age of 60 [26].

The stacking method proposed by Zhou et al. used sub-band and age features since slow-wave components vary significantly according to age [26]. Similarly, in [27], the authors used all the sub-bands mentioned above except K-complex and achieved an overall accuracy of 98.4% and 0.92 kappa using the random forest algorithm. The SVM-based sleep stage classification method proposed in [28] decomposed the signal data (EEG) into six sub-band (low−δ,high−δ,θ,α,σ,β) waves and local extrema. Then, statistical characteristics were extracted from each sub-band. The classifier produced 90.2% overall accuracy and 0.85 kappa.

## 3. Proposed Technique

**Figure 1:**
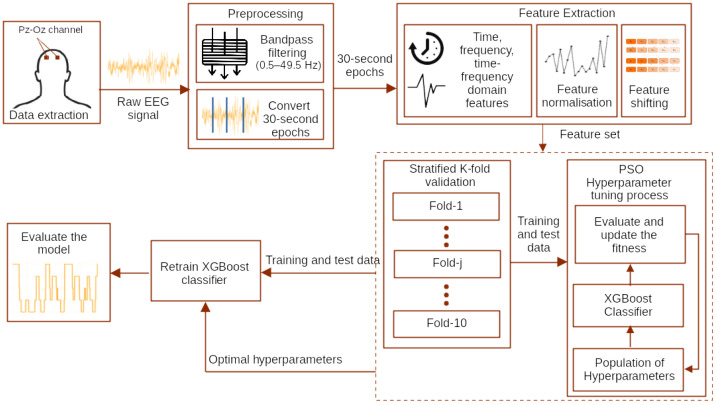
Proposed particle swarm optimisation combined with extreme gradient boosting (PSO–XGBoost) sleep-stage classification system architecture.

A reliable automated sleep stage classification system employing a single-channel EEG input is proposed in this work. Figure 1 depicts the schematic details of the proposed PSO–XGBoost system architecture. First, the input signal is filtered and segmented as 30 s epochs. Next, features are extracted from every 30 s epochs and the sub-band (δ, θ, α, σ, β, γ and K-complex) epochs. The participants’ age is considered as one feature because recent studies reveal that α signals and β signals significantly differ based on a subject’s age and gender [26,27]. Furthermore, in EEG signals, low frequencies decrease, and high frequencies increase for an age group of 20–60. Moreover, for ages greater than 60, low frequencies increase. The proposed system employs PSO-based hyperparameter optimisation to enhance sleep stage classification accuracy. The PSO-based hyperparameter optimisation is a one-time process that selects the best hyperparameter set for XGBoost. The steps for selecting the best hyperparameters are elaborated in Section 3.6. The proportion of sleep stages in the data is unequal; hence, it is an unbalanced classification. The class-balancing strategy is used to handle the unbalanced classification.

### 3.1. Data Extraction

This study used the EEG data from the public Sleep-EDF Expanded (SEDFEx) database. SEDFEx is a widely utilised database in the sleep research domain. Most of the proposed single-channel sleep classification systems in the literature used the SEDFEx database [16]. This study employed the SEDFEx database to fairly compare and validate the suggested approach to previous studies. This database possesses 197 EEG (Pz-Oz electrode and Fpz-Cz electrode), EOG (horizontal), chin EMG, and events marked by an event marker from whole-night sleep PSG recordings. Every participant recorded their two nights of sleep. Among the 197 recordings, 153 belonged to the sleep cassette (SC) experiment. The SC experiment participants had no sleep-related disorders. The remaining 44 recordings belonged to the sleep telemetry (ST) experiment. The ST experiment participants had mild sleep difficulty; hence, their sleep was recorded after temazepam and placebo intake. All the recordings were sampled at 100 Hz (i.e., 100 data points per second). Every recording was named using a pattern SC4ssNEO-PSG.edf or ST7ssNJ0-PSG.edf. In this pattern, ss represents the subject number, and N denotes the night of recording.

The sleep technicians manually scored whole sleep stages using the Rechtschaffen and Kales method and stored them in the *Hypnogram file [29,30]. Every recording was named using a pattern SC4ssNEO-Hypnogram.edf. These hypnograms (sleep stages) comprise distinct sleep stages like W, REM, N1, N2, N3, N4 and ? W represents wake; REM denotes rapid eye movement sleep; N1 to N4 signifies non-rapid eye movement (NREM) sleep; the symbol “?” illustrates ungraded epochs. The proposed method combined the NREM stages N3 and N4 as slow-wave-sleep (SWS) as per the American Academy of Sleep Medicine (AASM) [31] guidelines. Ten healthy subjects’ PSG recordings from a sleep cassette experiment that do not have unreported events and movements were used for the experiments; the summary of selected EEG recordings used in the proposed experiments is presented in Table 1. The number of total sleep stages in the selected recording was 10,600.

### 3.2. Pre-Processing

The proposed study selected the Pz-Oz EEG channel since it has been utilised extensively in the literature. This bipolar channel’s signal produced greater accuracy when compared with the Fpz-Cz channel [22,27]. An artefact like eye movement is quite common in recording electrodes positioned in the back of the brain. Further, a portion of γ waves during wake and sleep stages substantially impacts sleep stage classification [32,33]. Hence, the EEG data used in the experiments are filtered using a bandpass filter. The bandpass filter is configured with a hamming window and a lower and upper passband edge of 0.5 to 49.5 Hz [27]. Subsequently, the EEG recordings are converted into 30 s epochs without any overlap. Next, the stages N3 and N4 have been renamed as slow-wave stages (SWS) for all the used EEG recordings based on the AASM manual [31]. The excess W stages are trimmed at the start and end of each PSG file used.

All the participants in the SEDFx database are adults; hence, the α wave range is altered to 9–11 Hz. Again, the posterior head’s β wave falls between 13 and 20 Hz but gradually rises to 20 to 30 Hz during the REM stage. Therefore, the β wave is split into two parts: β1 wave (13–20 Hz) and β2 wave (20–30 Hz) to better distinguish between the W and REM stages [26]. Additionally, the γ1 wave is split into γ1 (30–40 Hz) wave and γ2 (40–49.5 Hz) wave based on the approach presented in [27]. They demonstrated that the γ wave significantly impacts the classification of sleep stages. This work agrees with earlier research [16] for the remaining wave ranges, which are recorded in Table 2. The data were normalised to mitigate the amplitude difference between different subjects.

### 3.3. Feature Extraction

It is a significant step for identifying essential features in data to enhance the performance of a machine learning algorithm. From the Pz-oz channel, 51 features were extracted from statistical, time, and frequency domains. The age of the participants was counted as one of the features because the α, β, and δ signals significantly differ concerning age. Finally, a total of 52 features were used for the experiments. In this work, X(m) denotes the Pz-Oz channel signal in a 30-s epoch with 3000 data points based on the sampling rate of 100 Hz. The Section 3.3.1, Section 3.3.2 and Section 3.3.3 describe the features used in this work.

#### 3.3.1. Time-Domain Features

The morphological properties can be represented using the features extracted from the time domain. The time-based characteristics extracted in our study are presented below.

Statistical features: Moments represent a set of statistical parameters that are used to measure any distribution. Time series moments of the first to fourth order mean (average), variance (how uniformly the values are distributed around the mean), skewness (represents the shape of a distribution), and kurtosis (determines whether a distribution is peaked or flat) initially extracted [16]. Their mathematical representation is given in Equations (1)–(4).

(1) $$\bar{x}=\frac{\sum_{i=1}^{i=M}x_{i}}{M}$$

(2) $$X_{var}=\frac{1}{M}\sum_{i=1}^{i=M}{\left(x_{i}-\bar{x}\right)}^{2}$$

(3) $$X_{skew}=\frac{\frac{1}{M}\sum_{i=1}^{M}{\left(x_{i}-\bar{x}\right)}^{3}}{{\left(X_{var}\right)}^{3/2}}$$

(4) $$X_{kurt}=\frac{\frac{1}{M}\sum_{i=1}^{M}{\left(x_{i}-\bar{x}\right)}^{4}}{X_{var}{\left(x\right)}^{2}}$$

Additional time-domain features: The additional statistical features, viz absolute additional time-domain features: The additional statistical features, viz absolute maximum value (AMV), root mean square (RMS), zero-crossing rate (ZCR), Hjorth mobility (HM), Hjorth complexity (HC), and detrended fluctuation analysis (DFA) are extracted. AMV is defined as the greatest possible point in every epoch. RMS measures the magnitude of a group of selected signals. The rate at which a signal transitions from positive to zero to negative and vice versa is known as the ZCR. Both RMS and ZCR are measured using Equations (5) and (6).

(5) $$X_{RMS}=\sqrt{\frac{1}{M}\sum_{i=1}^{M}x_{i}^{2}}$$

ZCR is defined as the rate of signal sign changes (positive to negative and vice versa); it can be defined as

(6) $$X_{ZCR}=\frac{1}{M-1}\sum_{i=1}^{M}1_{R<0}\left(x\left(m\right)x\left(m-1\right)\right)$$

where x(m) represents a signal size *M* and $1_{R<0}$ is the indicator function that is described as

(7) $$1_{R<0}\left(s\right):=\left\{\begin{array}{c} 1,\;\mathrm{if}\;s<1\\ 0,\;\mathrm{if}\;s\geq 0 \end{array}\right.$$

HM parameter represents the power spectrum’s mean frequency, and HC represents the frequency change [34]. Both HM and HC are computed using Equations (8) and (9).

(8) $$X_{HM}=\sqrt{\frac{X_{var}\left(\frac{dx}{dt}\right)}{X_{var}\left(x\right)}}$$

(9) $$X_{HC}=\sqrt{\frac{X_{HM}\left(\frac{dx}{dt}\right)}{X_{HM}\left(x\right)}}$$

The sequence connectivity of a signal is analysed by DFA. Then, we applied band-pass filters to separate δ (0.5–4 Hz), θ (4–8 Hz), α (9–11 Hz), σ (12–15 Hz), β1 (14–20 Hz), β2 (20–30 Hz), γ1 (30–40 Hz), γ2 (40–49.5 Hz), and K-complex (0.5–1 Hz) waves from each epoch. After dividing the nine sub-bands, the energy feature was calculated for every epoch using Equation (10). Similarly, the peak-to-peak amplitude was also extracted [27].

(10) $$X_{Energy}=\sum_{i=1}^{M}{\left|x_{i}\right|}^{2}$$

#### 3.3.2. Frequency-Domain (FD) Features

Power spectral density (PSD) explains how the signal’s power is spread over the frequency. The squared value of the signal is used to calculate the power and measured in energy per frequency. Similarly, PPA is estimated from every sub-band [35]. These features are versatile features that are used to describe variations in EEG data. The following are the features obtained from the nine sub-bands.

- Power features: ($\delta_{power}$, $\theta_{power}$, $\alpha_{power}$, $\sigma_{power}$, $\beta 1_{power}$, $\beta 2_{power}$, $\gamma 1_{power}$, $\gamma 2_{power}$ and $K-complex_{power}$).
- PPA features: ($\delta_{amp}$, $\theta_{amp}$, $\alpha_{amp}$, $\sigma_{amp}$, $\beta 1_{amp}$, $\beta 2_{amp}$, $\gamma 1_{amp}$, $\gamma 2_{amp}$ and $K-complex_{amp}$).

#### 3.3.3. Time-Frequency Domain (TFD) Features

Because EEG signals are non-stationary, their properties change over time. The following are the TFD features extracted.

Entropy and complexity feature: Entropy-based approaches measure the signal’s irregularity and impurity. The following are the entropy measures extracted entropy, permutation entropy (PE), spectral entropy (SPE), singular value decomposition entropy (SVDE), and sample entropy (SE). Equation (11) is used to obtain the entropy.

(11) $$X_{Entropy}=-\sum_{i=1}^{M}P_{i}Lm\left(P_{i}\right)$$

where $P_{i}$ represents the probability of the *i*th sample, and M is the count of samples [36]. PE provides a quantification measure of the complexity of an EEG signal by considering the order of relationships among the values of a signal and extracts a probability distribution from the EEG patterns. SPE measures the signal’s spectral power distribution. The SVDE quantifies the data’s dimensionality. SE estimates the randomness of EEG data without having any prior knowledge about it [16].

Next, the Lempel–Ziv complexity (LZC) and maximum and minimum distance (MMD) are extracted. LZC is utilised to evaluate the complexity of EEG signals [37]. MMD calculates the distance among the maximum and minimum signal samples in every subwindow, where $\Delta x_{m}$ and $\Delta y_{m}$ are the x-axis and y-axis deviations of the maximum as well as minimum signal samples in the *m*th window, respectively [38]. MMD is represented using Equation (12)

(12) $$X_{MMD}=\sum_{i=1}^{M}\Delta x_{m}^{2}+\Delta y_{m}^{2}$$

Fractal Features: The fractal dimension describes the behaviour of random-shaped signals by defining the measure of self-similarity on a given signal. This study included the Petrosian fractal dimension (PFD), Katz fractal dimension (KFD), Higuchi fractal dimension (HFD), and Hurst exponent (HE).

PFD turns a signal into a binary sequence and then calculates the fractal dimension [39]. The Equation (13) represents PFD.

(13) $$X_{PFD}=\frac{log_{2}^{M}}{log_{2}^{M}+log_{2}^{\left(M+0.4K\right)}}$$

KFD finds the distance (maximum) between any two points from the samples using the sum and average of the Euclidean distances in the consecutive data points of the signal [40]. KFD is represented in Equation (14).

(14) $$X_{KFD}=\frac{log_{2}^{M}}{log_{2}^{M}+log_{2}^{\left(d/L\right)}}$$

HFD stands for an approximation of the box-counting dimension of a signal graph. HE measures the time series’ long-term memory, i.e., the amount by which the series deviates from a random walk. The scalar denotes a time series’ relative proclivity to regress completely to the mean or a cluster in a particular direction [41].

The studies [26,42] show that EEG slow-wave components significantly differ based on the age group. Hence, the age feature is also incorporated into the feature set. Finally, a total of 52 features were extracted and are listed in Table 2.

#### 3.3.4. Feature Shifting

**Figure 2:**
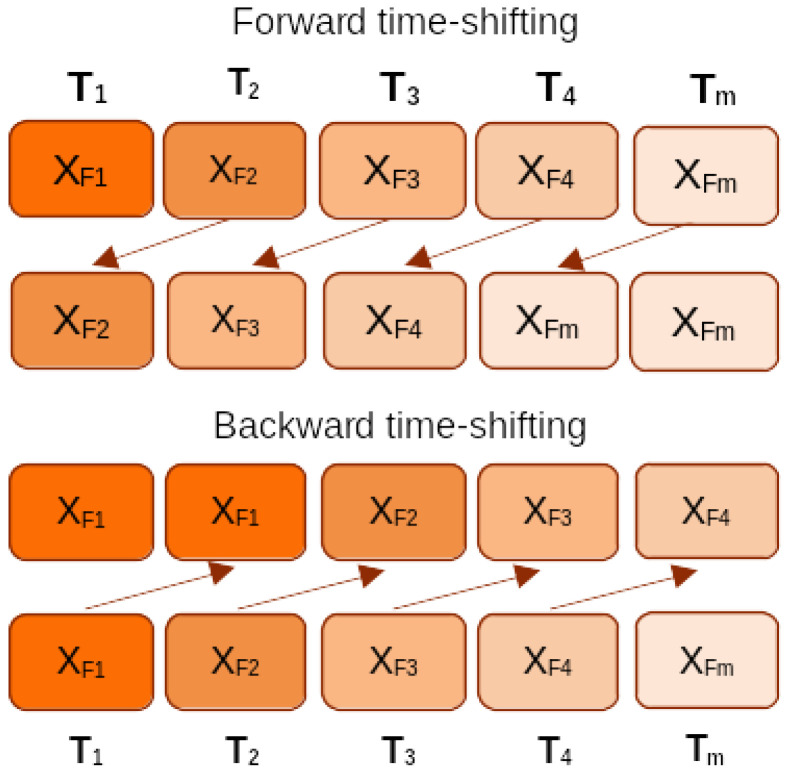
Backward and forward time-shifting of features.

The discrete data points in the EEG signals create a time sequence. That ordering generates a dependency between adjacent data points, giving more information to the classifier and improving prediction accuracy [43]. The studies [44,45] use this approach in deep learning models. This work takes a different approach to shift the features in time. The extracted 51 features are shifted one time-step forward and one time-step backward. Mathematically, let $S_{t}^{m}=\left\{X_{t-m},\ldots ,X_{m},\ldots ,X_{t+m}\right\}\in X_{m}$ is a sequence of 2*m* + 1 neighbouring data points, that is *m* data points from the past and *m* future data points. Distributing across every data point in $S_{t}^{k}$ aggregating 2*m* + 1 outputs creates a vector size of V(2*m* + 1). Finally, V(2*m* + 1) is fed into the classifier [43]. All the extracted 52 features, except ‘age’, are shifted forward and backward at one time. Eventually, the proposed model used 3 × 51 = 153 + 1 (age feature) = 154 features. Figure 2 shows this approach. And the impact of age future in the classification performance is shown in the Supplementary Materials. This approach boosted the overall accuracy by 3% to 4% without much computation.

### 3.4. XGBoost

XGBoost is a popular algorithm for classification tasks. The input $x_{j}$ is used to make the prediction ${\hat{y}}_{j}$, and it is represented in Equation (15).

(15) $${\hat{y}}_{j}=\sum_{m}\lambda_{m}x_{jm}$$

The hyperparameters are the undetermined parts that are unknown and need to be learned from data. XGboost contains three classes of hyperparameters, namely general parameters (GP), booster parameters (BP), and learning task parameters (LTP) [10]. The model training involves finding the best hyperparameter λ for the ${\mathrm{DS}}^{\left(tr\right)}$ and its labels $y_{j}$. The objective function given in Equation (16) measures how well the model fits ${\mathrm{DS}}^{\left(tr\right)}$.

(16) obj(λ)=L(λ)+Ω(λ)

where *L* is the loss during training, and Ω is the term that represents regularisation. The classification task here is a multi-class classification. Hence, ‘mlogloss’ given in Equation (17) is used as a choice of error for the classifier.

(17) $$L=-\frac{1}{M}\sum_{j=1}^{M}\sum_{k=1}^{N}y_{j,k}log\left(p_{j,k}\right)$$

here, *M* denotes the count of training samples from the dataset DS, $p_{j}$ indicates the prediction by XGBoost, $y_{j}$ represents the actual label, and *N* signifies the number of output classes. Regularisation helps to control the overfitting issue, and it is defined in Equation (18).

(18) $$\Omega \left(f\right)=\gamma T+\frac{1}{2}\lambda \sum_{i=1}^{T}\omega_{i}^{2}$$

where γ and λ represent tunable parameters, *T* signifies the number of leaves in a tree, and ω implies a score vector on the leaves. The XGBoost model optimises the objective function utilising an additive training procedure, i.e., the latter phase’s optimisation method relies on the preceding stage’s outcomes [10,14]. The XGBoost model’s *t*th objective function can be redefined by considering multi-class log loss (17) and regularisation (18) into (16).

(19) $$obj^{\left(t\right)}=L^{\left(t\right)}+\Omega \left(f_{t}\right)+C$$

where $L^{\left(t\right)}$ represents the ‘mlogloss’ term of the *t*th round’, $\Omega (f_{t})$ represents the regularisation at the *t*th round’, and C is the constant term.

### 3.5. Particle Swarm Optimisation (PSO)

PSO is one of the population-based optimisation approaches inspired by bird flocking. In each iteration, until the ultimate optimum is detected, every particle in a swarm interacts with different particles to identify and revise its current global optimum [46]. PSO has been demonstrated to be efficient and robust in the diverse optimisation of real-world problems except for sleep stage detection [47]. For machine learning models, choosing the appropriate hyperparameter configuration directly impacts the model’s performance [48]. Hyperparameter tuning using PSO comprises these elements:

- Tunable hyperparameter λ, ($\lambda^{1},\lambda^{2},\ldots ,\lambda^{n}$);
- Dataset DS;
- Model M;
- Score function F.

The parameter tuning job entails optimising a hyperparameter λ so that the model M trained in a dataset DS with hyperparameters *P* maximises some score function F. This is represented in Equation (20).

(20) maxλF(λ),F(λ)=score(M≥(DS))

To estimate the best hyperparameter configuration, accuracy is used as a scoring function.

### 3.6. PSO–XGBoost Classification

**Figure 3:**
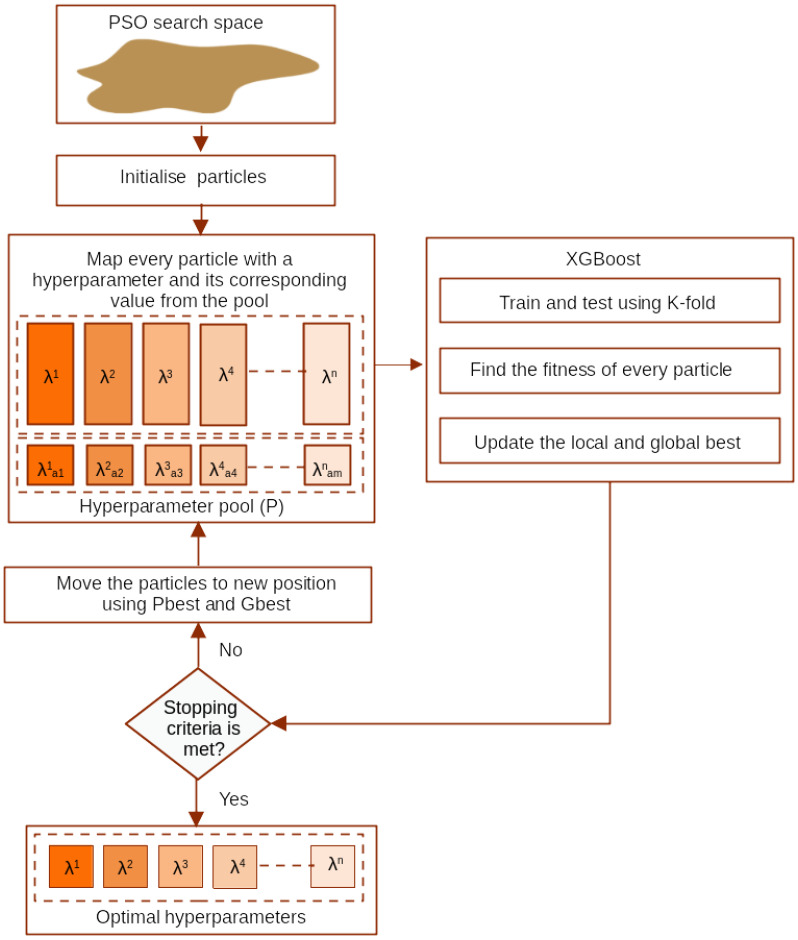
Tuning extreme gradient boosting (XGBoost) hyperparameters using particle swarm optimization (PSO).

This section describes the proposed PSO–XGBoost method for single-channel EEG signal classification. This study optimises the XGBoost classifier’s accuracy using PSO to estimate the best hyperparameters (from the GP and BP classes) as shown in Figure 3.

Let λ be the hyperparameter set representing every possible hyperparameter of a model M. λ is partitioned into multiple subsets $\lambda =\{\lambda^{1},\lambda^{2},\ldots ,\lambda^{n}\}$, where n is the number of hyperparameters needed to train M. For every $\lambda^{j}$, where 1 ≤ *j* ≤ *n*, distinct possibilities related to the same hyperparameter type are available for selection. One value is usually selected from every subset and assigned to M. M is trained and accuracy is measured.

In the PSO-based approach, the complete list of hyperparameter combinations refers to the search space. Particles (referred to as individuals) are navigating into hyperdimensional exploration space. The change of particle positions inside the exploration space depends on the victory of other particles. Therefore, changes in the position of particles inside the swarm are inspired by its neighbouring particles’ expertise. Moreover, the exploration activities of a particle are influenced by other particles inside the swarm. As a result, creating that social behaviour can be referred to as a stochastic process, i.e., particles return towards earlier successful regions in the swarm space. The possibilities count in search space is the product of the number of values in every hyperparameter group $\prod_{j=1}^{m}z_{j}$, where $z_{j}$ is the size of hyperparameter subset $\lambda^{j}$ [49,50]. The search process is continued till meeting termination criteria. All the particles traverse in *S* till achieving the termination criterion [14,51,52]. The detailed algorithm for the best hyperparameter estimation using PSO is as follows.

1. The search space for PSO is initialised. It is bounded by the maximum and minimum values of the hyperparameters. The search space is *n*-dimensional with *n* as the number of hyper-parameters to be optimised.
2. Inside the search space, *m* particles are generated randomly. Each particle is a vector of length *n*. Every particle *k* in the exploration space (search space) *S* has a position (location) (P) and velocity (V) characteristics. The position describes a set of hyperparameters $\left(P_{k}\right)$ of M, and the velocity defines every particle’s traverse path in the search space over every dimension (hyperparameter). $P_{k}$ is depicted as $P_{k}$ = $\{P_{k}^{1}$, $P_{k}^{2}$, …, $P_{k}^{n}\}$, where $P_{k}^{d}$ represents the position of the particle in a dimension *d*, 1 ≤ *d* ≤ n, n is the number of dimensions. $V_{k}$ is represented as $V_{k}$ = $\{V_{k}^{1}$, $V_{k}^{2}$, …, $V_{k}^{n}\}$, where $V_{k}^{d}$ refers to the velocity of the particle in a dimension *d*, 1 ≤ *d* ≤ *n*.
3. The inertia and learning (cognitive and social) parameters of PSO are set. The velocity vector of each particle is set to zero. The best positions of the individual particles (local best) are assigned as current positions.
4. For each particle in the set, the model M is trained and accuracy is measured. The particle with high accuracy is assigned as the global best particle. The best positions of the individual particles are updated based on their corresponding model accuracy. In a particle search space, each particle contains information regarding the local best ($P_{lb}$) and global best ($P_{gb}$) position. In each iteration, every particle is compared to its $P_{lb}$ and $P_{gb}$. On finding a better solution, the $P_{lb}$ and ∥ or $P_{gb}$ are updated.
5. The velocity of each particle is updated. Then particles are moved to new positions through the updated velocity.
  (21) $$V_{kd}^{j+1}=\omega V_{kd}^{j}+c1r1\left(P_{lb_{kd}}^{j}-P_{kd}^{j}\right)+c2r2\left(P_{gb_{kd}}^{j}-P_{kd}^{j}\right)$$
  (22) $$P_{kd}^{j+1}=P_{kd}^{j}+V_{kd}^{j+1}$$
  where $V_{kd}^{j}$ represents the jth particle’s velocity in *d* during the iteration *j*. $P_{kd}^{j}$ denotes the jth particle’s position in *d* during iteration *j*. $P_{lb_{kd}}^{j}$ indicates the achieved position of the particle in *d* during iteration *j*. $P_{gb_{kd}}^{j}$ specifies the best solution achieved in the search space till the jth iteration in *d*. The other variables, ω, r1 and r2, c1 and c2 are inertia weight, random numbers and social and cognitive parameters, respectively.
6. The process is repeated from Step 4 until the stopping criterion is met. In this work, the maximum number of iterations has been selected as the stopping criterion.
7. The global best particle gives the best hyper-parameter set for the model (M).

## 4. Experiment

### 4.1. Experimental Setup

The entire experiments of this paper were implemented and tested using the Python 3.6.3 environment. The MNE Python library was used to accomplish preprocessing and converting the data into 30 s epochs [53]. The Optunity Python was used to perform hyperparameter optimisation for PSO implementation [54].

The model’s training employs the subject-independent strategy to emphasise the significance of generalisation and model robustness. After combining all the subject data listed in Table 1, features were extracted for processing. The features extracted from the EEG signal were divided into training and testing data using a stratified-k-fold CV to assess the model’s performance. It ensures training and test sleep stages have the same proportion of the stages as available in the dataset. Implementing a target variable provides the cross-validation outcome, a near approximation of generalisation error. In our experiments, the value of k was set to ten; hence, the data was split into ten parts (nine parts used for training and the one left used for testing) [55]. During the data stratification, every fold included the balanced proportions of five classes (W, N1, N2, SWS, and R), which helps to avoid selecting a specific training and testing test. Two experiments were carried out. The default hyperparameter setting of the XGBoost classifier was employed in the first experiment. Next, the second experiment used the PSO-optimised hyperparameters.

The XGboost’s hyperparameters classes GP and BP were tuned to put our proposed approach into action. GP relates to the type of booster used, and we considered ‘gbtree’ and ‘dart’ based on the objective (multiclass classification). BP depends on the type of booster selected. The PSO uses all the parameters related to the tree booster. While using the ‘dart’ booster, XGBoost uses dropouts, leading to evaluating some trees alone; hence, ‘ntree_limit’ was used in the predict function. LTP decides the learning environment. The LTP parameters ‘objective’ and ‘eval_metric’ correspond to the learning objective. For ‘objective’, we used ‘multisoftmax’ (multiclass) and its mandatory parameter ‘num_class’ was set to 5 (count of output classes). For ‘eval_metric’, we used ‘mlogloss’ during training–testing and ‘multisoftprob’ was used for the ROC curve. In order to design the best fitness function, classification accuracy was considered. Therefore, the particle having a better fitness value produces good accuracy during classification [14,51,52].

### 4.2. Performance Evaluation

Indeed, observing the classification performance on a validation set is an excellent way to obtain feedback on the developed classifier. It is also an invaluable tool for comparing two distinct models. Our ultimate goal is to create a better, more accurate model that assists in making better decisions in real-world scenarios. Researchers widely adopted the stratified K-fold cross-validation (CV) technique in multi-class classification to estimate the error rate. The data were arbitrarily partitioned into ten balanced proportions (*K* = 10), with each class represented in roughly the same proportions as the complete dataset. As a result, the learning operation was repeated ten times on distinct training sets. Eventually, an overall error estimate was calculated by averaging the ten error estimates. It enables us to lower the bias rate with random samples. The CV accuracy ($CV_{Acc}$) implies the k individual accuracies [55].

(23) $$CV_{Acc}=\frac{1}{K}\sum_{j=1}^{K}Acc_{j}$$

where *K* represents the count of folds, and $Acc_{j}$ describes the calculated accuracy in each fold.

In a multi-class classification scenario, metrics like accuracy or precision/recall do not give us a complete view of the classifier’s performance. Cohen’s kappa (κ) statistic is an excellent metric for handling multi-class and imbalanced-class issues [56,57].

(24) $$\kappa =\frac{P_{o}-P_{e}}{1-P_{e}}$$

The observed agreement is $P_{o}$, whereas the projected agreement is $P_{e}$. It essentially informs how well the classification algorithm performs compared to a classifier that guesses at random depending on the frequency of every class. Kappa is never greater than or equal to one. As per the Landis et al. method, the κ value of 0 shows no agreement, κ range 0.21–0.40 indicates fair, κ range 0.41–0.60 represents moderate, κ range 0.61–0.80 indicates substantial, and κ range 0.81–1 signifies a nearly ideal agreement [56]. The receiver operating characteristic (ROC) study is a primary tool for diagnostic accuracy in clinical medicine, and assesses and presents all possible combinations of specificity and sensitivity. The area under the curve (AUC) is a performance criterion for issues in classification at diverse threshold levels. The degree of separability is explained by AUC, while ROC explains the probability curve. It shows how well the model can differentiate across classes [58].

### 4.3. Experiment 1: Classification Using Default Hyperparameters

**Figure 4:**
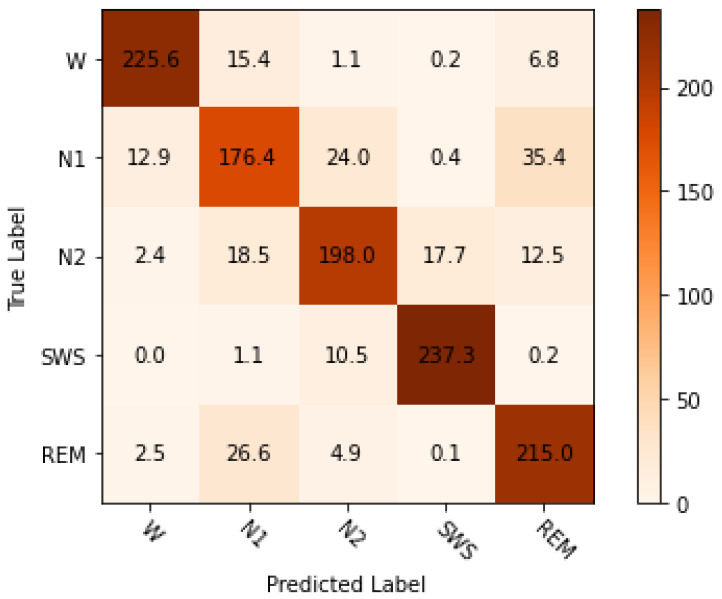
Extreme gradient boosting (XGBoost) mean confusion matrix using stratified 10-fold cross-validation (CV) (without optimal hyperparameters).

**Figure 5:**
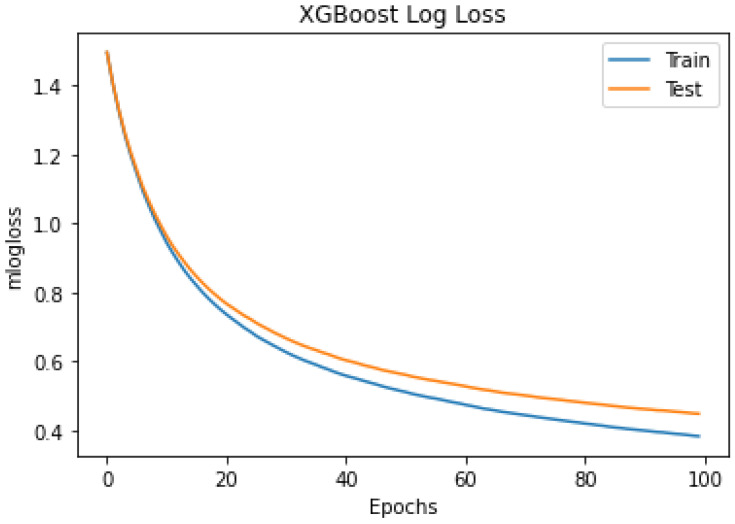
Extreme gradient boosting (XGBoost) multi-class logloss using stratified 10-fold cross-validation (CV) (without optimal hyperparameters).

**Figure 6:**
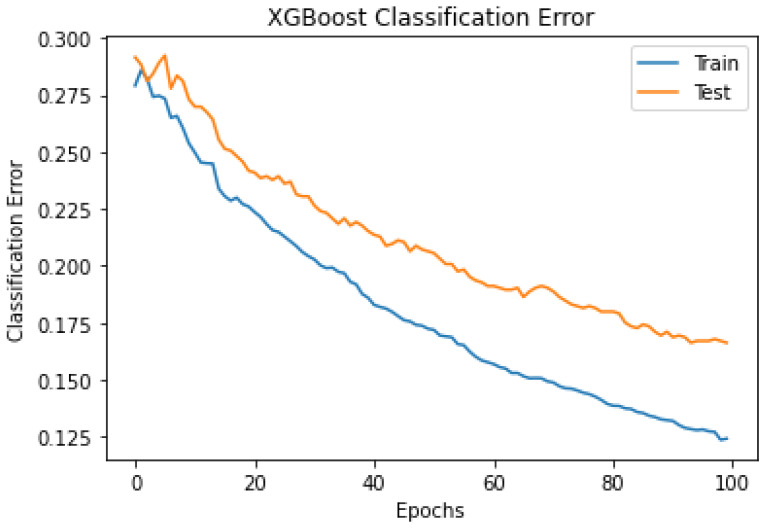
Extreme gradient boosting (XGBoost) multi-class classification error using stratified 10-fold cross-validation (CV) (without optimal hyperparameters).

**Figure 7:**
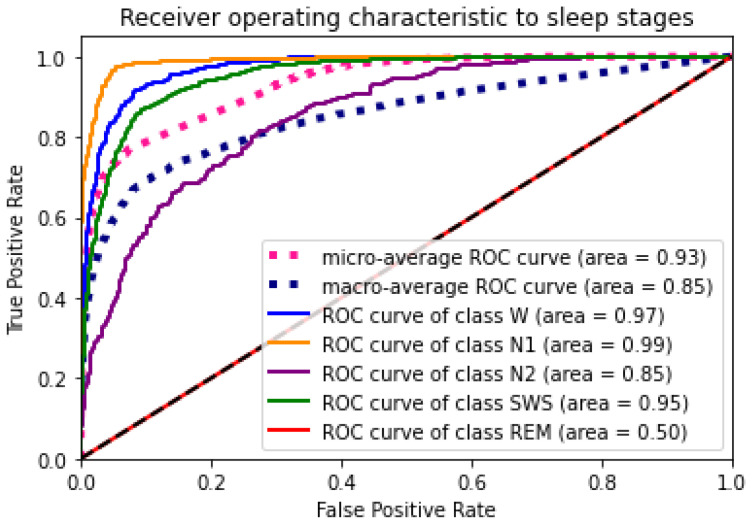
Extreme gradient boosting (XGBoost) receiver operating characteristic to sleep stages (without optimal hyperparameters).

This experiment used the XGBoost model with the default hyperparameter values except for ‘objective’ and ‘$num_{c}lass$’. The ‘objective’ and ‘num_class’ parameters used ‘multi:softmax’ and five, respectively, based on the target classes count. The classifier’s performance was measured using stratified K-fold CV, prediction accuracy, f1-score, precision, recall, and kappa. Table 3 shows the hyperparameter set and the default values used in the experiment. The ‘booster’ parameter values can be ‘gbtree’ and ‘dart’. Both boosters ‘gbtree’ and ‘dart’ were tested separately. The ‘dart’ booster achieved a higher accuracy level than gbtree. When using ‘gbtree’ as a booster, it uses a bunch of additional parameters presented in Table 3. This experiment considered the default values for the additional parameters related to the ‘dart’ booster. As shown in Table 4, the XGBoost classifier achieved a mean, maximum, and minimum accuracy of 84.5%, 84.9%, and 83.4%, respectively. This experiment achieved an accuracy level of some existing research works that used the sleep-EDFx repository’s Pz-Oz channel [19,22]. Additionally, the achieved classification accuracies provide a reference to improve the capability of the proposed PSO–XGBoost model. The mean confusion matrix from the 10-folds are presented in Figure 4. Figure 5 represents the multi-class logloss of the classifier during training and testing. The classifier’s error during training and testing is presented in Figure 6. Finally, the class-wise accuracy between different sleep stages is depicted in Figure 7 using the receiver operating characteristic graph.

In the default hyperparameter experiment, even though the classifier achieved decent results, it suffers from classification errors. Figure 6 and Figure 7 show that the classifier failed to draw a correct boundary between classes. Hence, there is an increased classification error between sleep stages. Figure 4 shows the misclassification rate between sleep stages (W, N1, N2, SWS, and REM). When classifying sleep stage W, the classifier ends up classifying as N1 (15.4), N2 (11), and REM (6.8). The misclassification rate between W and N1 is high compared to the other sleep stages. Similarly, the classifier faces issues in detecting sleep stage N1 from N2 (24) and REM (35.4); here, error percentage is very high. Additionally, the classifier encounters an issue when classifying N2 with N1 (18.5), SWS (17.7), and REM (12.5). Again, when classifying SWS with N1 (11) and N2 (10.5), the error percentage is less. Finally, the misclassification count between REM vs. N1 (26.6) is very high. Even though the classifier is trained with the essential features extracted from different domains, it fails to make a clear boundary between the classes. It can be improved by creating an optimal model with suitable hyperparameters.

### 4.4. Experiment 2: Classification Using PSO Optimised Hyperparameters

**Figure 8:**
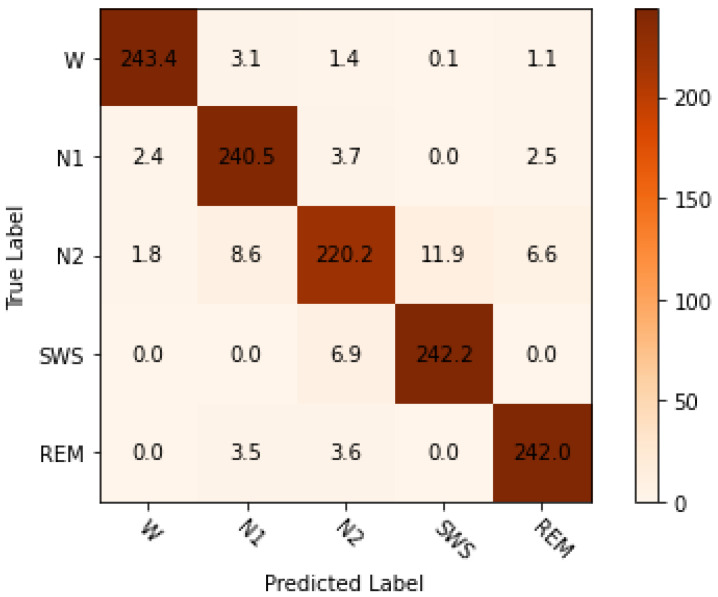
Particle swarm optimization combined with extreme gradient boosting (PSO–XGBoost) mean confusion matrix using stratified-10-fold cross- validation (CV) (with optimal hyperparameters).

**Figure 9:**
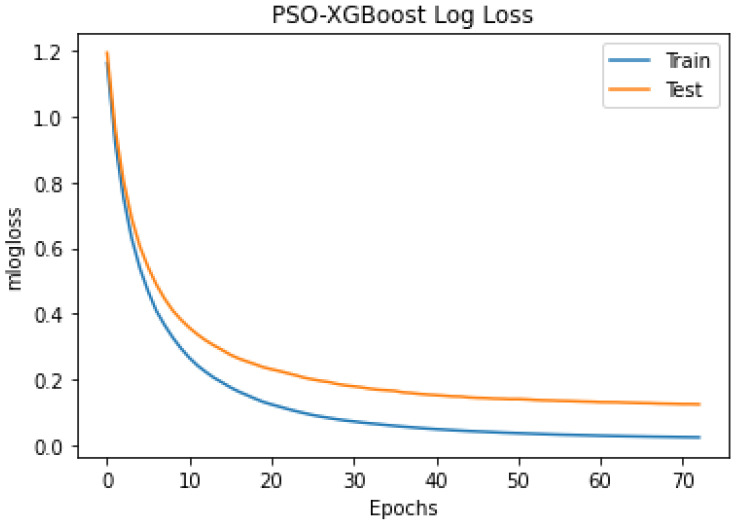
Particle swarm optimization combined with extreme gradient boosting (PSO–XGBoost) multi-class logloss of using stratified-10-fold cross- validation (CV) (with optimal hyperparameters).

**Figure 10:**
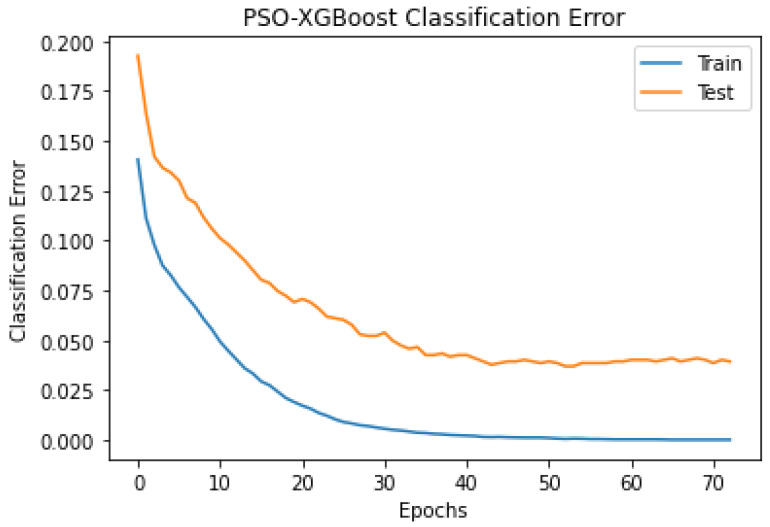
Particle swarm optimization combined with extreme gradient boosting (PSO–XGBoost) multi-class classification error of using stratified-10-fold cross- validation (CV) (with optimal hyperparameters).

**Figure 11:**
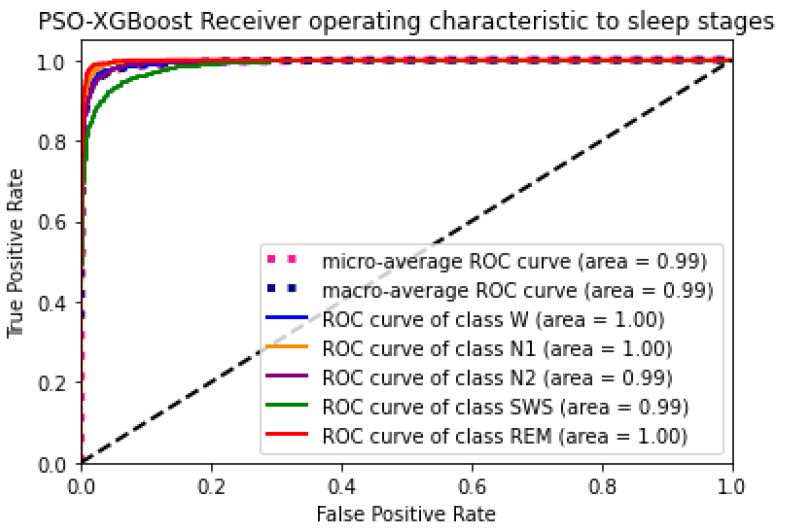
Particle swarm optimization combined with extreme gradient boosting (PSO–XGBoost) receiver operating characteristic to sleep stages (with optimal hyperparameters).

The proposed PSO–XGBoost classification approach improves the XGBoost classification method by automatically optimising the hyperparameters using PSO. This experiment aims to find the effectiveness of this methodological improvement using the sleep-EDFx repository’s Pz-Oz channel. The training dataset is utilised for training the proposed PSO–XGBoost classifier. The PSO–XGBoost classifier’s accuracy is tested with the test samples at the end of the optimisation procedure. The achieved average accuracy is 95.4%, which is an increase in accuracy over the XGBoost classifier’s output (using a ‘dart’ booster). The PSO–XGBoost results are shown in Table 5; the classifier achieved mean, maximum, and minimum accuracies of 94.0%,95.0%, and 93.0%, respectively. The results exhibit the PSO–XGBoost classifier’s ability to subdue the gap between the lower and the better class accuracy while preserving high overall accuracy. The mean confusion matrix from the 10-folds is presented in Figure 8. Figure 9 represents the multi-class logloss of the classifier during training and testing. The classifier’s error during training and testing is presented in Figure 10. Finally, the class-wise accuracy between different sleep stages is depicted in Figure 11 using the receiver operating characteristic graph. In the optimal hyperparameter experiment, Figure 10 and Figure 11 show significant improvement in the error rate and classification error. Similarly, Figure 8 shows that the classifier has improved its decision boundary between the sleep stages. After tuning the model with optimal hyperparameters, the misclassification rate reduced between W and N1 (3.1), N2 (1.4), and REM (1.1). Similarly, the misclassification error decreased between N1 and N2 (3.7), SWS (0), and REM (2.5). Here, the misclassification rate of SWS becomes zero, which is a considerable achievement. The misclassification rate declined between N2 with N1 (8.6), SWS (11.9), and REM (6.6). Again, when classifying SWS with N1 (0), N2 (6.9), and REM(0), the error percentage reduced a lot. Finally, the misclassification count between REM vs. W (0), N1 (3.5), N2 (3.6), and SWS (0.0) has dropped when compared with experiment 1. The experiment 2 results reveal a significant improvement in overall accuracy and a notable reduction in error rate. Tuning the model parameters using PSO positively impacts the results and creates a better model.

According to sleep-stage classification literature [16], a considerable amount of work either implemented upsampling or downsampling of the whole dataset to balance sleep class categories. This approach procedure might produce less generalisation ability and overfitting. Even though this approach improves performance to some degree, it is improper. This work upsampled only the training data to address the class imbalance problem. It could assist the classifier in improving the accuracy of less-represented sleep stages.

The minute inter-class difference is always common in the EEG signals. The PSO–XGBoost approach used the advantage of adjacent epoch information with the current epoch using feature shifting. It facilitated the classifier to detect classes more accurately. The confusion matrix Figure 8 clearly visualises it.

As stated in Section 2, some studies implemented the signal decomposition approaches such as EMD [17,18,19], TQWT [23,24], and the hybrid approach [25]. Though these works achieved decent classification results, they are computationally intensive. The PSO–XGBoost approach conducts the signal decomposition using sub-bands. It can be achieved using a digital filter and is computationally less intensive. Also, recent works demonstrated that extracting features from the sub-bands contributes significantly to a better classification outcome [26,27,28].

The outcomes of the experiments (Table 4 and Table 5) present the mean accuracy, f1-score, precision, recall, and kappa using the stratified 10-fold CV from XGBoost and PSO–XGBoost. According to the nature of the classification task, the multi-class logloss played a vital role in choosing the best model. PSO–XGBoost subdued logloss, as shown in Figure 9, which is lesser than those obtained using the default parameter values represented in Figure 5. PSO–XGBoost reduced the validation errors in Figure 10 that are lesser than the validation errors when using default hyperparameter values in Figure 6. Although the XGBoost with default hyperparameters showed better classification prediction performance for the sleep-EDFx dataset, the accuracy rate is not fair enough. Therefore, the proposed PSO–XGBoost is capable of providing a solution with minimised error rates and logloss. In our experiments, PSO presented more optimal parameter values for XGBoost. The classification summary of both XGBoost with default hyperparameters and PSO–XGBoost is presented in Figure 4 and Figure 8. Figure 8 clearly shows that the percentage of false negatives significantly reduced after optimisation.

Generally, for the larger dataset, when the search space is more extensive, the computational cost of PSO is high. Because it requires training and testing multiple classifiers, PSO’s fitness function becomes slow. However, this can be solved with distributed PSO.

The performance achieved by the ‘dart’ booster method for classifying sleep stages rests in the following: First, the input features are extracted for classification. Second, the most relevant features derived from the EEG signal are based on the processing method. The characteristic patterns of EEG in varying frequency ranges have a high correlation with sleep stages. For this specific reason, features were also extracted from the signal sub-bands δ (delta), θ (theta), α (alpha), σ (sigma), β1 (beta-1), β2 (beta-2), γ1 (gamma-1), γ2 (gamma-2), and K-complex. It allows the design of more accurate single-channel sleep monitoring systems for automatic sleep stage detection [26,27].

Accurate identification of the sleep stage from an EEG signal is essential for diagnosis and medication. The proposed PSO–XGBoost model classifies the sleep stages from an EEG signal with a mean accuracy level of 95.4%. The AU–ROC curve counts on the PSO–XGBoost classifier’s performance in a classification task. This result improved the ROC AUC and F-measure of PSO–XGBoost; it achieved better accuracy than the existing classification methods listed in Table 6. The mean κ value and κ value from each fold during experiment 1 and experiment 2 are presented in Table 4 and Table 5. Table 5 reveals an increased agreement level in PSO–XGBoost. Also, the proposed PSO–XGBoost achieved a better κ value among the existing works [17,18,19,20,21,23,24,25,26,27,28], which as shown in Table 6.

The proposed PSO–XGBoost method helps to create a detection strategy based on repeating patterns. It can also be utilised on data collected after the primary classification system has been developed. The extracted features are robust enough to detect different sleep stages beyond the classifier’s training patterns. However, there is small discrimination in detecting the correct N2 stage from N1 and SWS since these stages have a shallow difference (Figure 8). In terms of estimating the prediction error using a 10-fold CV, the EEG data set shows that a 10-fold CV error has negligible bias. The estimated bias was little and further reduced with more training samples. Although the features are generated independently of sleep stages, a classifier with an average zero error and an average CV error near zero is possible.

Database variability is a common concern in medical research and development. For instance, two distinct systems have simultaneously monitored identical physiological variables. Hence, the data produced by both systems would differ due to the typical signal-to-noise ratio. In light of this, handling data from one of the two systems may be challenging for a learning model that has only been exposed to examples from the other. For this reason, an ML model’s generalization abilities should be assessed in a more expansive scenario, specifically by considering two or more independent data sources concerning a single task, with the data being kept separate from the model’s development and parameterisation process. To solve this problem, the proposed models’ performance is evaluated separately using different databases. Table 7 depicts the performance of proposed approaches in other databases. Ten records from each database have been used for this experiment.

### 4.5. Model Deployment

There is tremendous growth in home-based health systems [3]. Home-based devices are capable of delivering several advantages to a diverse user group. For example, in-home sleep monitoring can be employed in remote locations or rural areas where it is more difficult to access traditional healthcare providers. This might result in fewer hospital trips or primary care providers, saving expenditures. Also, continuous monitoring is feasible at a lower cost in a home environment. The senior generation and rural people may benefit the most from this technology.

**Figure 12:**
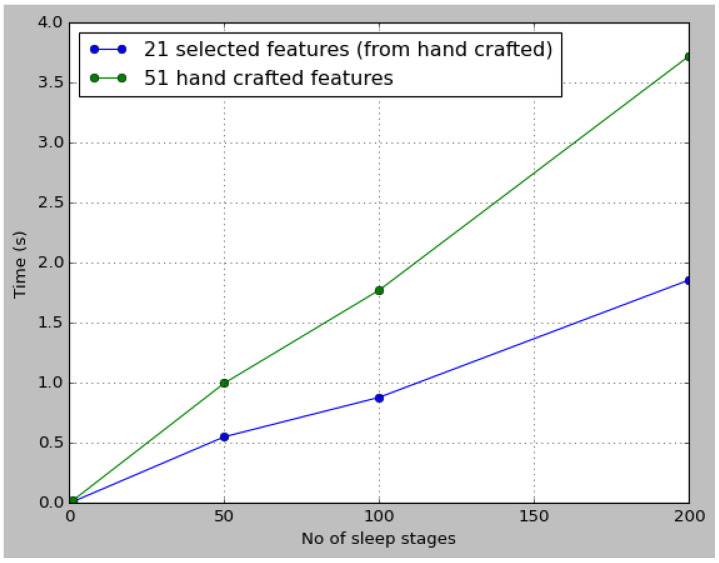
mRMR selected vs. handcrafted features extraction time in Raspberry Pi.

We have deployed the PSO–XGBoost classifier on the Rasberry Pi-3 B model to verify its ability to generate features for the test data and classify sleep stages. Figure 12 depicts the variation in feature extraction time for different numbers of epochs using with and without feature selection. The mRMR feature selection approach selected 21 of the 51 features applying the mRMR technique. As depicted in Figure 12, the processing time increased in Rasberry Pi as the epochs increased. The proposed model’s post-deployment accuracy and kappa measures slightly reduced accuracy from 1% to 2%. This evaluation was performed using only SEDFx data.

## 5. Conclusions

Enhancing sleep-stage detection accuracy is a critical issue in automated sleep-stage classification systems. Multi-class classification issues like sleep-stage classification can effectively harness the power of PSO–XGBoost. This study presented an efficient automated sleep-stage detection approach using a PSO–XGBoost model. The Pz-Oz channel data of the SEDFx repository is used to visualise a single-channel EEG system. Features are extracted from every 30-s epoch and nine sub-bands from the Pz-Oz raw EEG signal. Next, the features are shifted in time (one step forward and backwards) to include the temporal relation between data points. A sleep-stage classification model is built using XGBoost. Then, PSO is employed to explore the optimal hyperparameters adaptively. It outperformed the existing approaches when compared with the overall classification performance. The proposed model has the following advantages. It just needs a single-channel EEG. PSO–XGBoost’s overall classification performance facilitates clinicians in precisely detecting and monitoring sleep stages. The feature shifting process is not computationally intensive and contributed to the overall accuracy improvement of 3% to 4%. The significant advantage of the PSO–XGBoost model is that it can support resource-constrained real-time in-home sleep monitoring systems. This study’s shortcoming is that to improve generalisation, the model needed to be extensively trained using data from several databases.

## Figures and Tables

**Table 1:** List of selected subject recordings.

Subject	Recordings	Age	Sex	Record Length
0	SC4001E0, SC4002E0 *	33	Female	22:05:00
2	SC4012E0	26	Female	23:45:00
3	SC4031E0	26	Female	23:30:00
5	SC4051E0	28	Female	22:41:00
6	SC4061E0	31	Female	23:05:00
11	SC4011E0	26	Male	23:21:00
12	SC4012E0	26	Male	23:45:00
13	SC4131E0	27	Male	23:27:00
18	SC4182E0	28	Male	23:41:00

* Two recordings selected from the same subject.

**Table 2:** List of extracted features.

Features			
1	Age	27	θ 4–8 Hz
2	mean	28	α 9–11 Hz
3	median	29	σ 12–15 Hz
4	absolute maximum value	30	β1 14–20 Hz
5	variance	31	β2 20–30 Hz
6	skewness	32	γ1 30–40 Hz
7	kurtosis	33	γ2 40–49.5 Hz
8	root mean square	34	0.5–1 Hz K-complex
9	peak-to-peak amplitude	35	power of δ
10	entropy	36	power of θ
11	permutation entropy	37	power of α
12	spectral entropy	38	power of σ
13	singular value decomposition entropy	39	power of β1
14	sample entropy	40	power of β2
15	Katz fractal dimension	41	power of γ1
16	Higuchi fractal dimension	42	power of γ2
17	number of zero-crossings	43	power of K-complex
18	detrended fluctuation analysis	44	amplitude of δ
19	Hurst exponent	45	amplitude of θ
20	maximum minimum distance	46	amplitude of α
21	Lempel–Ziv complexity	47	amplitude of σ
22	Hjorth Mobility	48	amplitude of β1
23	Hjorth complexity	49	amplitude of β2
24	power spectral density	50	amplitude of γ1
25	Petrosian fractal dimension	51	amplitude of γ2
26	δ 0.5–4 Hz	52	amplitude of K-complex

**Table 3:** Hyperparameters of extreme gradient boosting (XGBoost) (Experiment1).

Hyperparameter	Default Configuration
base_score	0.5
booster	‘ *gbtree*’, ‘*dart*’
colsample_bylevel	1
colsample_bynode	1
colsample_bytree	1
gamma	0
learning_rate	0.1
max_delta_step	0
max_depth	3
min_child_weight	1
n_estimators	100
num_class	5
objective	‘multi:softmax’
random_state	0
reg_alpha	0
reg_lambda	1
scale_pos_weight	1
subsample	1
sample_type *	‘*uniform*’, ‘*weighted*’
normalize_type *	‘*tree*’, ‘*forest*’
rate_drop *	0.0
one_drop *	0
skip_drop *	0.0

*—additional parameter used by ‘dart’ booster.

**Table 4:** Performance of extreme gradient boosting (XGBoost) using stratified-10-Fold cross-validation (CV) (without optimal hyperparameters).

Fold	Accuracy	F1-Score	Precision	Recall	Kappa
1	0.845	0.844	0.846	0.845	0.806
2	0.848	0.847	0.848	0.848	0.810
3	0.834	0.832	0.832	0.834	0.792
4	0.857	0.856	0.857	0.857	0.821
5	0.849	0.849	0.850	0.849	0.811
6	0.843	0.843	0.843	0.843	0.803
7	0.856	0.856	0.857	0.856	0.820
8	0.847	0.846	0.847	0.847	0.808
9	0.836	0.836	0.836	0.836	0.795
10	0.834	0.834	0.835	0.834	0.792
Mean	0.845	0.844	0.845	0.845	0.806

**Table 5:** Performance of particle swarm optimization combined with extreme gradient boosting (PSO–XGBoost) using stratified-10-fold (with optimal hyperparameters).

Fold	Accuracy	F1-Score	Precision	Recall	Kappa
1	0.957	0.956	0.957	0.957	0.946
2	0.959	0.959	0.959	0.959	0.949
3	0.963	0.963	0.963	0.963	0.954
4	0.951	0.951	0.951	0.951	0.939
5	0.945	0.945	0.946	0.945	0.932
6	0.952	0.952	0.952	0.952	0.940
7	0.964	0.964	0.964	0.964	0.955
8	0.944	0.943	0.944	0.944	0.930
9	0.945	0.945	0.946	0.945	0.932
10	0.961	0.961	0.961	0.961	0.951
Mean	0.954	0.954	0.954	0.954	0.943

**Table 6:** Performance of the particle swarm optimization combined with extreme gradient boosting (PSO–XGBoost) and existing approaches.

Dataset: Sleep-EDF, Channel: Pz-oz				
Author	Year	Classifiers	5-Class %	Kappa
Hassan [17]	2016	Adaboost	90.1	-
Hassan [18]	2016	Bagging	90.6	-
Hassan [24]	2016	Randiom Forest	91.5	0.861
Hassan [19]	2017	RUSBoost	83.4	0.840
Hassan [23]	2017	Bagging	94.3	0.943
Seifpour [28]	2018	SVM	90.2	0.870
Jianga [21]	2019	Random Forest	88.3	0.817
Ghimatgar [22]	2019	Random Forest	81.8	0.740
Zhou [26]	2020	Stacking	91.2	0.864
Liu [20]	2021	XGBoost	92.2	0.877
Zeo [25]	2022	Bagging	89.3	-
Proposed Work	-	PSO–XGBoost	95.4	0.943

**Table 7:** Performance of proposed approach in SEDFx, DREAMS, and SHHS.

Proposed Approach	Database	Channel	5-Class %	Kappa
PSO–XGBoost	Sleep-EDFx	Fpz-Cz	92.1	0.90
PSO–XGBoost	Sleep-EDFx	Pz-Oz	95.4	0.94
PSO–XGBoost	DREAMS	Cz-A1	80.3	0.75
PSO–XGBoost	SHHS	C4-A1	78.8	0.74

## Data Availability

The data used to support the findings of this study are available from the corresponding author upon request.

## Supplementary Materials

The following supporting information can be downloaded at: https://www.mdpi.com/article/10.3390/s24041197/s1, Table S1: Performance of particle swarm optimization combined with extreme gradient boosting (PSO–XGBoost) using stratified-10-fold (with optimal hyperparameters) using age feature; Table S2: Performance of particle swarm optimization combined with extreme gradient boosting (PSO–XGBoost) using stratified-10-fold (with optimal hyperparameters) without age feature.

## Abbreviations

The following abbreviations are used in this manuscript:

PSO	Particle swarm optimisation
AASM	American Academy of Sleep Medicine
EEG	Electroencephalogram
REM	Rapid eye movement
NREM	Non-rapid eye movement
